# Three Cases of Infantile Apoplexy

**Published:** 1858-01

**Authors:** Joseph H. Wythes

**Affiliations:** Port Carbon, Pennsylvania


### ART. IV.—*Three cases of Infantile Apoplexy.* By JOSEPH H. WYTHES, M.D., of Port Carbon, Pennsylvania.

AMONG medical writers, apoplexy occurring under twenty-one years of age is said to be of such rare occurrence that I find but few cases on record. Some authors, indeed, scarcely admit its existence during infancy; yet, judging from the cases which have fallen under my own observation, I am inclined to believe it to be not so uncommon as has been supposed.

Meningeal congestion and hemorrhage are admitted to be sometimes met with at an early age, but, as Dr. Wood remarks, "It is not characterized by the usual apoplectic phenomena, and ranks, by the symptoms, rather with meningitis." Cerebral hemorrhage, on the other hand, is so rare, that Dr. Bennet doubts whether the two cases recorded by M. Lallemand and M. Billard were cases of apoplexy; and Dr. J. F. Meigs says that "Rilliet and Barthez met with only eight cases in their extensive experience, and that M. Barrier saw but one in 576 cases of disease of all kinds."

It has several times happened in the practice of the writer, to meet with cases of paralysis, more or less general, in children from two to six years old, occurring suddenly, and in no practical sense differing from the same affection following an attack of apoplexy in an adult. In the instances recorded below, death appeared to have taken place instantaneously, while the *post-mortem* examinations left no doubt but that the immediate cause of dissolution was the excessive and sudden accumulation of blood in the cerebral vessels. In the second of the following cases, dissection revealed the existence of meningitis, as well as extensive disease of the substance of the brain itself; yet the total absence of premonitory symptoms, and the suddenness and overwhelming nature of the fatal attack, have caused me to regard it as a case of apoplexy occurring in a brain already diseased, and from that cause more likely to give way.

The excessive amount of gas in the intestines in each of these cases is a symptom which may not be unsuggestive, although I am not prepared to attach any special value to it; yet it was in greater quantity than is usually observed in other examinations at a similar age.

CASE I.—On the evening of May 31st, 1852, I was called to see the child of Mr. Shaefer, about three years old, who was complaining of pain in the left ankle. It was not swollen or discolored, but the pain seemed excessive, especially on motion. The parents supposed it to have sprained its ankle while playing on the step. I ordered a stimulating liniment, and, as it had been costive, a dose of castor oil.

The next morning I was informed that the child had been found dead, lying in the bed between its parents, without awaking either. On examination I found the wrists and ankles of the child strongly contracted; the left leg, the arms, and the back considerably discolored with dark purple blood; and the left leg slightly swollen. As it was the wish of the family to have a *post-mortem* examination, I called in Dr. James Parker to assist me.

On removing the calvarium, which was done with as little violence as possible, an extravasation of blood was observed between the cranium and dura mater, perhaps half a fluid-ounce. The vessels of the membranes were much injected, and the posterior part of the septum had a large clot of blood in it. The membranes were also in many places strongly adherent. The white substance of the brain was full of bloody dots or points, with the appearance of infiltrated blood in the posterior lobes, particularly on the right side. The right ventricle had in it a clot of blood, with serum.

The viscera of the chest appeared healthy. The liver was much enlarged, and contained a few cysts. The stomach was disproportionably small. The appearance of the intestines was generally healthy, but about one-half of the ileum was distended with gas. A few tubercles were observed in the mesentery.

CASE II.—Nov. 4th, 1855, was called to see the child of S. Landis, who had died suddenly. I officiated at its birth exactly three months previously. It was a vigorous male child, and had always been in good health, with the exception of occasional attacks of colic, for which no medical attendance was deemed necessary. On Saturday night, Nov. 3d, the family retired to bed at 12 o'clock, and the child seemed as usual. At 5 or 6 o'clock in the morning the mother laid him off her arm, as she supposed, asleep. After breakfast, wondering he slept so long, she went to rouse him, and found him dead.

When I first saw him, about 10½, A.M., he was quite cold. The muscles of the hands and feet were quite contracted, but particularly so on the left side; the mouth was drawn slightly awry; and the whole posterior surface from head to foot was of a purple hue. I suspected cerebral disease, and asked for a *post-mortem* examination, which was granted. I was assisted by my brother, Dr. Wm. W. Wythes, Dr. G. Brown, and in the latter stages by Dr. J. S. Carpenter,—the last-named gentleman having arrived too late to inspect the brain.

The vessels of the arachnoid and pia mater were considerably congested, and the whole cerebral substance was softened, being about the consistence of melted butter. A little serum was observed at the base of the left hemisphere of the cerebrum, but nothing abnormal in the ventricles. The membranes of the cerebellum and medulla oblongata were strongly congested.

The lungs, heart, and abdominal viscera were quite healthy, though the latter were fully distended with gas.

CASE III.—Dec. 20th, 1855, was called to make a *post-mortem* examination of the child of J. R., aged three months, which had been found dead in its bed. Was assisted by Dr. G. Brown.

Muscles of hands and feet much contracted. Foam issuing from mouth and nose. Face purple. Purple congestion of the skin of the back and limbs, probably from gravitation.

The membranes of the brain healthy, but its substance much congested. On slicing, it seemed full of bloody dots. A small clot of blood was found in each ventricle. Lungs congested. A serous effusion observed in the pericardium. Liver large, and intestines full of gas. Stomach and bowels otherwise healthy.

